# Three-dimensional velocity vector image obtained via 4-dimensional flow magnetic resonance imaging for in-stent flow visualization in the superficial femoral artery

**DOI:** 10.1016/j.radcr.2022.12.055

**Published:** 2023-01-17

**Authors:** Mitsunari Maruyama, Hiroya Aso, Hisatoshi Araki, Rika Yoshida, Shinji Ando, Megumi Nakamura, Takeshi Yoshizako, Yasushi Kaji

**Affiliations:** Department of Radiology, Shimane University Faculty of Medicine, P.O. Box 00693-8501, 89-1 Enya cho, Izumo, Japan

**Keywords:** Four-dimensional flow magnetic resonance imaging, Stent, Endovascular therapy

## Abstract

The assessment of stent lumen patency via non–contrast-enhanced 2-dimensional time-of-flight magnetic resonance angiography (2D TOF MRA) is complex due to stent-related artifacts. However, an imaging technique using the phase-contrast method, which can reduce susceptibility to artifact, is available. Herein, we report the use of 3-dimensional velocity vector image obtained via 4-dimensional flow magnetic resonance imaging (4D flow MRI) for in-stent flow visualization after stent development in the right superficial femoral artery. Hence, instead of 2D TOF MRA, 4D flow MRI using the phase-contrast method can be performed to assess stent lumen patency as it reduces stent-related artifacts.

## Introduction

Four-dimensional flow magnetic resonance imaging (4D flow MRI) can be performed for the volumetric and time-resolved visualization and quantification of blood flow [1]. The target vessel flow velocity measurement, wall shear stress, and other parameters can be analyzed. Four-dimensional flow MRI is clinically applied for the assessment of diseases in different body systems, including the cerebral arterial, cardiovascular, and portal venous systems [2], [3], [4]. However, it is utilized for evaluating lower extremity arterial diseases.

Non–contrast-enhanced magnetic resonance angiography (MRA) is useful for detecting lower extremity arterial stenosis in cases wherein contrast media cannot be used due to reasons such as renal dysfunction and contrast media allergy. Stent imaging and assessment of stent lumen patency via MRI are significantly complex due to stent-related artifacts. The severity of such artifacts is based on stent characteristics, such as stent material and design, and imaging parameters [5]. An imaging technique using the phase-contrast method, which reduces susceptibility to artifact, is available [5,6]. Herein, we report the use of 3-dimensional (3D) velocity vector image obtained via 4D flow MRI for in-stent flow visualization after stent development for right superficial femoral artery (SFA) occlusion.

## Case report

### Endovascular therapy procedure

**Fig. 1:**
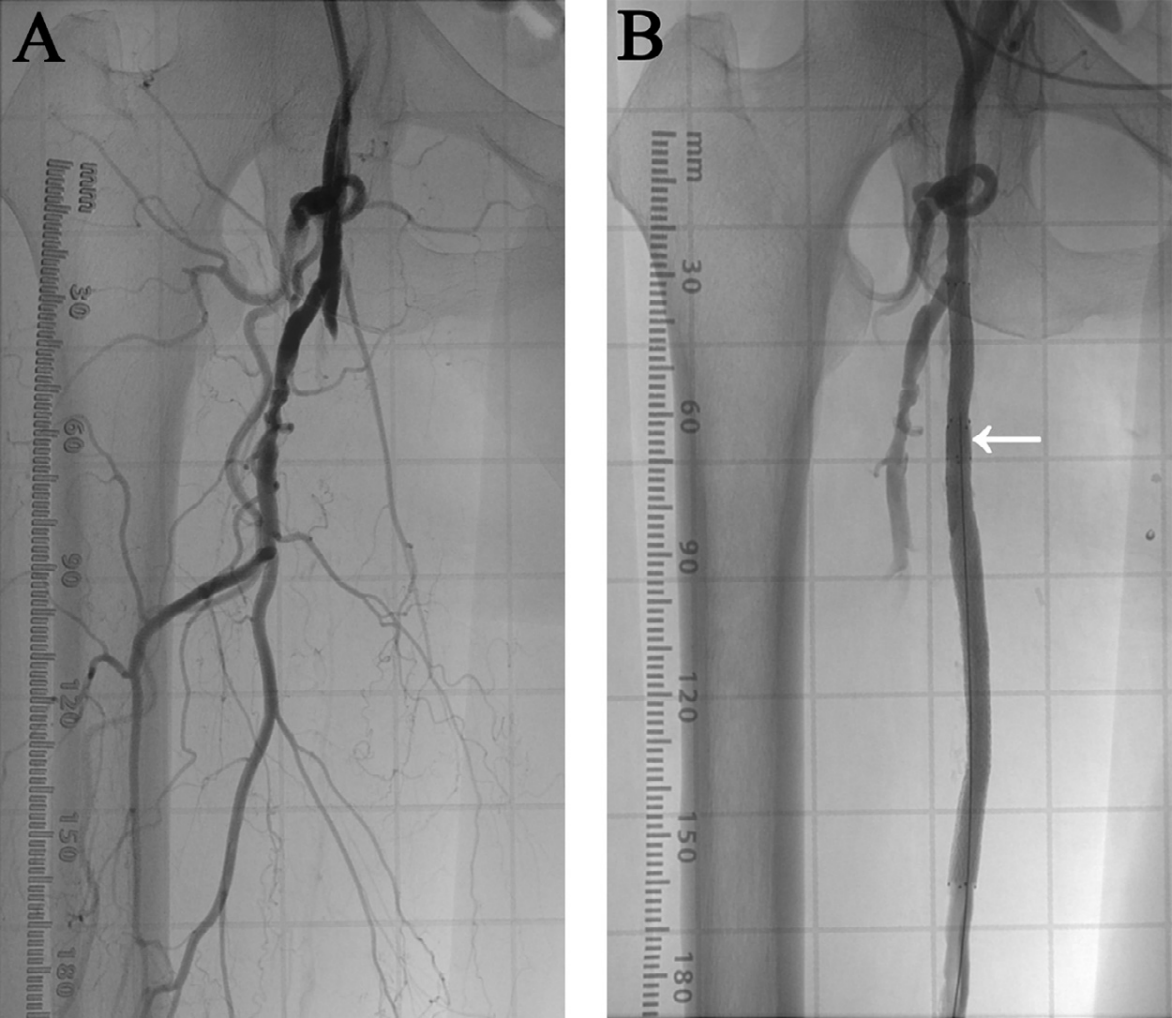
Digital angiography (DA) images of the right superficial femoral artery (SFA). (A) DA image before endovascular therapy. Right SFA occlusion was observed. (B) DA image after stenting. Stent deployment was performed using a fluoropolymer-coated paclitaxel-eluting stent (Eluvia; Boston Scientific Japan, Tokyo, Japan). Revascularization in the right SFA was observed. There was no stenosis at the stent-in-stent site (arrow).

A 70-year-old woman presented with intermittent claudication of the right lower limb (Rutherford category 3) [7]. The initial non–contrast-enhanced 2-dimensional time-of-flight magnetic resonance angiography (2D TOF MRA) showed the location of atherosclerotic lesions that could be effectively treated with endovascular therapy (EVT). Results showed stage II (FP3IP0) right SFA occlusion classified using the Global Limb Anatomical Staging System [8]. Vascular access was achieved percutaneously by establishing an antegrade right common femoral artery puncture. Right SFA occlusion was observed on digital angiography imaging (Fig. 1A). Vessel preparation was performed using a balloon (Sterling; Boston Scientific Japan, Tokyo, Japan), and stent deployment was performed using a fluoropolymer-coated paclitaxel-eluting stent (Eluvia; Boston Scientific Japan, Tokyo, Japan). Stent-in-stent development was performed because the stent length was insufficient for the lesion length. Stenting was successful, and Figure 1B shows the digital angiography image after EVT. Significant hemodynamic improvement was observed after EVT, and the right ankle-brachial index increased from 0.40 to 0.97.

### Two-dimensional TOF MRA

The patient underwent 2D TOF MRA and 4D flow MRI on day 1 after EVT. MRI was performed using a 3T MRI scanner (Ingenia Elition X, Philips Medical Systems, Best, the Netherlands). Signal excitation and detection were conducted using a standard quadrature RF birdcage body coil and a 20-channel head/neck coil, respectively. ECG-gated non–contrast-enhanced 2D TOF MRA was performed with the following parameters: TR/TE/flip angle: 5.8 ms/3.5 ms/50°; k-space segmentation/temporal resolution: 1 ms/83 ms; field of view/matrix: 336 × 336 mm/336 × 384 (recon matrix); slice thickness/number of slices: 3.5 mm/−0.5 gap; and voxel size: 1.19 × 1.19 × 3.50 mm^3^. The approximate acquisition time was 2.60 min (1 stack).

### Three-dimensional velocity vector image obtained via 4D flow MRI

For 4D flow MRI acquisitions, a retrospectively ECG-gated, time-resolved, 3D, phase-contrast MR sequence with referenced 3-directional velocity encoding was used. Four-dimensional flow MRI of the whole femur was conducted with the following parameters: oblique axial orientation—TR/TE/flip angle: 4.9 ms/3.0 ms/12°; k-space segmentation/temporal resolution: 4 ms/78 ms; field of view/matrix: 350 × 350 mm/241 × 135 (recon matrix 320); slice thickness/number of slices: 3.0 mm (gap −1.5)/70; voxel size: 1.68 × 3.01 × 3.00 mm^3^; velocity encoding (VENC): maximum velocity of distal external iliac artery × 1.2 cm/s; and compressed SENSE: × 6. The VENC was set during scan prescription, and it adjusted the velocity encoding gradients such that the maximum velocity can be measured via phase-contrast MRI without velocity aliasing. The approximate acquisition time was 2.56 minutes. Data were reconstructed to 10 time frames per R-R intervals.

Four-dimensional flow MRI imaging was performed using an image analysis system (SYNAPSE VINCENT; Fujifilm Medical, Tokyo, Japan). Three-dimensional velocity vector image was obtained via 4D flow MRI, and 3D velocity vector image was compared to 2D TOF MRA image after stent development.

**Fig. 2:**
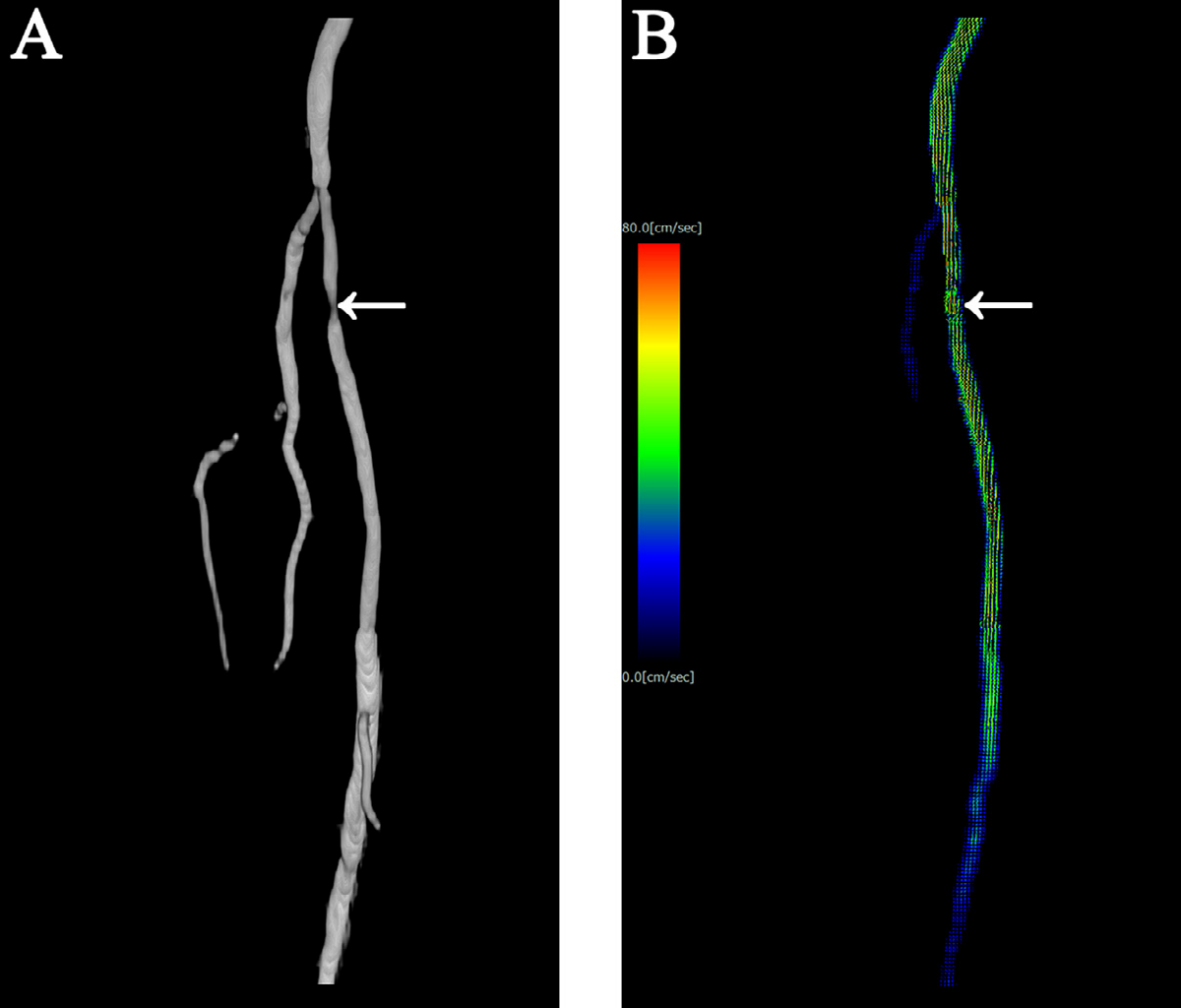
Two-dimensional time-of-flight magnetic resonance angiography (2D TOF MRA) and 3-dimensional (3D) velocity vector image obtained via 4-dimensional flow magnetic resonance imaging (4D flow MRI) after stent development. (A) 2D TOF MRA. The stent lumen patency on 2D TOF MRA was complicated by stent-related artifacts. The stent lumen was more stenotic than the digital angiography (DA) image (Fig. 1B). Moreover, there was a high number of artifacts at the site where stent-in-stent was performed (arrow). (B) 3D velocity vector image obtained via 4D flow MRI. In-stent flow could be visualized on 3D velocity vector image obtained via 4D flow MRI, such as the DA image (Fig. 1B). Three-dimensional velocity vector imaging showed neither accelerated jet flows nor in-stent stenosis at the stent-in-stent area unlike in 2D TOF MRA (arrow).

The stent lumen patency on 2D TOF MRA was complex due to stent-related artifacts (Fig. 2A). There was a high number of artifacts at the site where stent-in-stent was performed (Fig. 2A, arrow). In-stent flow visualization could be achieved using 3D velocity vector image obtained via 4D flow MRI (Fig. 2B). Further, 3D velocity vector image showed neither flow acceleration nor in-stent stenosis at the stent-in-stent part (Fig. 2B, arrow).

## Discussion

Herein, we report the application of 3D velocity vector image obtained via 4D flow MRI for in-stent flow visualization after stent development, even though the stent lumen patency on 2D TOF MRA was complicated by stent-related artifacts. Eluvia (Boston Scientific Japan, Tokyo, Japan) is a self-expanding Nitinol stent with tantalum as the radiopaque stent marker. Pseudostenosis at the stent-in-stent site was caused by susceptibility to artifacts caused by radiopaque markers. An imaging technique using the phase-contrast method, which reduces artifact susceptibility, is available [5,6]. A shortened TE (ms) can suppress the effects of phase dispersion and can reduce susceptibility to artifacts caused by metals [9]. The TE (ms) of 4D flow MRI sequence using the phase-contrast method was shorter than that of 2D TOF MRI, and susceptibility artifact could be reduced. In a previous study, the assessment of stent lumen patency on 4D and 2D phase-contrast flow imaging was superior to conventional 3D contrast-enhanced MRA. Further, flow-based estimates were less affected by stent orientation and were less dependent on the stent material examined [5].

Four-dimensional flow imaging has not been applied for assessing lower extremity arterial diseases, and it is useful if contrast media should be avoided due to reasons such as renal dysfunction and contrast media allergy. Patients with significant stenosis for which EVT is indicated commonly present with a peak systolic velocity ratio of >2. Thus, it is possible to detect vascular significant stenosis by assessing flow acceleration on 4D flow MRI images. To identify significant stenosis that must be treated, the VENC should be high such that it is twice the flow velocity of the target vessel. VENC was set at the maximum velocity of the distal external iliac artery × 1.2 in this case. However, further research is required to determine the optimal VENC for SFA lesions. The dual-VENC method might be useful [10].

The pixel size for the target vessel was set at ≤30%, and spatially averaged velocity measurements were highly accurate. Moreover, maximum velocity could be measured with a high accuracy when the pixel size ratio was set at ≤10% [11]. Although the pixel size for the target vessel was large in this case, in-stent flow visualization, jet flow detection, and acquisition time shortening were prioritized. Compressed SENSE can shorten scan time and improve clinical efficiency [12]. In addition, the acquisition time in this case was approximately 3 minutes for 4D flow MRI using compressed SENSE. In-stent stenosis is challenging to detect via 2D-TOF-MRA alone. However, it can be identified by routinely combining 2D-TOF-MRA and 4D-flow-MRI, which can be acquired in minutes.

## Conclusion

In-stent flow was visualized using 3D velocity vector image obtained via 4D flow MRI. Thus, instead of 2D-TOF-MRA, 4D flow MRI can be performed for evaluating stent lumen patency as it reduces stent-related artifacts.

## Author contributions

All authors provided substantial contributions to the manuscript and approved the final version of the article to be published.

## Patient consent

Informed consent was obtained for the publication of this case report.
